# Management of SARS-CoV-2 Transmission in Emergency Dental Settings: Current Knowledge and Personal Experience

**DOI:** 10.1017/dmp.2020.483

**Published:** 2020-12-22

**Authors:** Marius Negucioiu, Alexandru Bucur, Ondine Lucaciu, Andrada Soanca, Alexandra Roman

**Affiliations:** 1 Department of Prosthodontics, Faculty of Dental Medicine, Iuliu Haţieganu University of Medicine and Pharmacy, Cluj-Napoca, Romania; 2 Emegency County Clinical Hospital, Cluj-Napoca, Romania; 3 Department of Oro-Maxillo-Facial Surgery, Faculty of Dental Medicine, University of Medicine and Pharmacy “Carol Davila”, Bucharest, Romania; 4 Spitalul Clinic de Chirurgie Oro-Maxilo-Faciala “Prof. Dr. Dan Theodorescu” Oro-Maxillo-Facial Clinical Hospital, Bucharest, Romania; 5 Department of Oral Health, Faculty of Dental Medicine, Iuliu Haţieganu University of Medicine and Pharmacy, Cluj-Napoca, Romania; 6 Department of Periodontology, Faculty of Dental Medicine, Iuliu Haţieganu University of Medicine and Pharmacy, Cluj-Napoca, Romania

**Keywords:** COVID-19, dental services, SARS-CoV-2 transmission, oral health

## Abstract

The coronavirus disease 2019 (COVID-19) has seen a violent and fast spread worldwide. The severe acute respiratory syndrome coronavirus 2 (SARS-CoV-2) has a predominantly respiratory transmission through droplets and aerosol with serious implications for dental settings. This article is based on recent research, guidelines issued by relevant authorities, as well as on the authors’ experience acquired through their involvement in setting up an emergency dental care hub in Cluj-Napoca, Romania, during the COVID-19 lockdown. The present article aims to provide a brief description of COVID-19 implications in dental office and to recommend preventive protocols for dental practitioners to ensure a safe and healthful workplace. The recommendations for infection control presented in this article address the specific risks of exposure to SARS-CoV-2. The article provides a special customized guideline covering patient triage and entrance into the dental practice, personnel protection, dental treatment, and after-treatment management. The implementation of strict preventive measures has been found to be efficient in the prevention of SARS-CoV-2 contamination because no infections have been reported among our staff or patients. COVID-19 is a major emergency worldwide marked by a rapid evolution and warranting a need for further assessment of the implications of COVID-19 outbreak in dental practice.

During this pandemic circumstance, we all witnessed the terrible expansion of severe acute respiratory syndrome coronavirus 2 (SARS-CoV-2) infection and its dramatic consequences. The respiratory disease deriving from SARS-CoV-2 infection was named coronavirus disease 2019 (COVID-19).^1^ Symptoms of COVID-19 may vary from fever and dry cough to nonspecific symptoms, such as sore throat, shortness of breath, conjunctivitis, diarrhea, vomiting, fatigue, and muscular pain.^2,3^ More recently, the loss of smell (anosmia) and taste (dysgeusia)^4^ as well as oral manifestations^5^ and cutaneous lesions^6^ have been reported. Severe complications include respiratory distress syndrome, arrhythmia, and shock.^2^ Many SARS-CoV-2 positive patients have demonstrated mild or no symptoms, which makes the clinical diagnosis challenging during the incubation period and, therefore, facilitates an accelerate spread of infection.^7^

Although community spread of the disease has somewhat decreased during the lockdown period, currently, the situation is far from being contained. On the contrary, COVID-19 has seen a violent and fast spread worldwide. There are over 34 million declared COVID-19 cases and over 1 million deaths globally at 1 October 2020.^8^

Dental professionals are at especially high risk of contracting the COVID-19 due to the unique nature of dentistry.^9^ As the COVID-19 crisis seems to prolong, the entire management of dental settings should focus on delivering safe conventional dental treatments to mitigate its impact on the patient’s oral health status. Reports from dental emergency settings indicate an increase in extractions and a decrease in endodontic treatment acceptance in patients presenting with more severe disease, possibly due to the clinical and economic consequences of the COVID-19 crisis.^10^ An increased number of antibiotic prescriptions before referral to the emergency clinics, sometimes incongruent with official recommendations,^11^ has also been reported.^10^

The spread of infection requires effective long-term infection control protocols to be implemented in dental settings to prevent SARS-CoV-2 contamination of medical personnel and patients. Dentists should use a combination of algorithms and teledentistry for patient triage and evaluation, as well as adapted treatment and organizational protocol for patient safety and minimal occupational hazards.^12^ In addition to the latest research on COVID-19 and to the various opinions produced by clinicians, public health officials, and the authorities, it is essential to assemble the data into utile protocols to run a long-term reliable dental clinical practice.

This article is based on recent research, guidelines issued by relevant authorities, as well as on the authors’ experience acquired through setting up an emergency dental care hub in Cluj-Napoca, Romania. The purpose of the present article is to offer a brief description of SARS-CoV-2 transmission, its implications for dental offices and to recommend preventive, ready-to-use protocols for dental practitioners. The recommendations presented in this article aim to provide an update of the already existing infection control plans of dental settings enabling the assessment of the specific exposure risks and of the other unique characteristics of SARS-CoV-2 transmission, as well as to offer guidance to dental personnel in providing a safe and healthful workplace.

## SARS-CoV-2 Mode of Transmission

Based on recent findings, it appears that the major route of transmission of SARS-CoV-2 is by means of respiratory secretions in the form of large respiratory droplets or splatters containing viral particles, produced when an infected person coughs, sneezes, or talks, propagated at a short distance, rather than by means of small aerosols.^13-15^ Splatter was defined by Micik and colleagues as airborne particles larger than 50 μm in diameter.^16-18^ These particles are too large to become suspended in the air and are airborne only briefly before contacting the floor.^19^

Aerosolized viral particles by cough, sneeze, or dental care procedures can potentially travel across greater distances, up to 6 m,^9^ and can remain suspended in the air for an extended period of time before they settle on environmental surfaces.^16-18^ Aerosols are formed by particles less than 50 μm in diameter.^16-18^ The nose typically filters air particles above 10 μm.^20^ The particles that are less than 10 μm can enter the respiratory system, whereas smaller particles can reach the alveoli.^20^ However, even if it is potentially possible, infection through small aerosolized particles is not the main route of SARS-CoV-2 transmission.^20,21^

Contact transmission through contaminated surfaces is also possible,^14^ but it is not considered a main route of transmission either. It has been reported that SARS-CoV-2 can persist on various surfaces for a few hours up to several days depending on environmental conditions.^21^

The virus persists up to 72 h after application to plastic and stainless-steel surfaces, up to 24 h on cardboard surfaces, and is viable up to 3 h in suspended aerosols.^22^ The fecal-oral and vertical transmissions of SARS-CoV-2 are about to be confirmed.^21,23,24^

Patients with symptomatic disease have been the main source of transmission,^13^ but asymptomatic carriers should also be considered as a potential source of infection.^25,26^

A dose-dependent clinical manifestation between SARS-CoV-2 load on contact and severity of the disease has been suggested. Patients with severe COVID-19 had a 60 times higher nasopharyngeal viral load than the patients with mild disease.^27^

However, despite the research findings summarized above, we need to agree that “COVID-19 is a new disease, and we are still learning about how it spreads and the severity of illness it causes”.^13^

## Contamination Pathways in Dental Settings

The potential routes for SARS-CoV-2 transmission in dental settings are contact with airborne infectious particles, direct contact with body fluids of an infected patient, and contact with contaminated environmental surfaces.^19^ Recently, To et al. (2020)^28^ recognized saliva as a reservoir of SARS-CoV-2 in infected individuals. In these circumstances, dental professionals appear at high risk of contagion.^15^

Most dental procedures use mechanical instrumentation and, thus, produce airborne particles.^19,29^ The ultrasonic scaler has been shown to produce the greatest amount of airborne contamination, followed by the air-driven high-speed handpiece, the air polisher, the air-water syringe, and the prophylaxis angles.^30-34^

A true aerosol or droplet nuclei may remain airborne in the dental practice for up to 30 min after a dental procedure,^29^ which means that immediate removal of the respirator/mask exposes the practitioner to a potential contact with airborne contaminated material.^19^

Preoperative antimicrobial mouth rinse reduces the number of bacteria generated by means of dental procedures.^35^ Hydrogen peroxide vapor has been shown to be virucidal (>4-log reduction) against some experimental viruses including a SARS-CoV surrogate,^36^ which suggests that 1.5% peroxide preprocedural rinsing could diminish oral and pharyngeal viral load. Preprocedural rinses could reduce, but not eliminate, the extent of contamination in dental practice because antiseptic rinses do not penetrate subgingivally. Consequently, this will not affect blood coming directly from the operative site or microorganisms harbored in the nasopharynx. While the use of the rubber dam is a standard protective gesture in restorative and endodontic procedures, it is not feasible for periodontal hygiene and surgical procedures. Periodontal procedures are always performed in the presence of blood and rely mostly on the use of ultrasonic scalers, which create the greatest amount of aerosol contamination.^19,37^ One recommended method to remove airborne contamination is the ultraviolet (UV) system, but it is cost prohibitive for most dental offices^19^ and proof of efficacy against SARS-CoV-2^13^ has not yet been reached. It seems more practical to take immediate measures to remove airborne contamination in the proximity of the generation site. The use of a high-volume evacuator reduces aerosol contamination from the operative site by more than 90%.^19,31^ In addition, during aerosol-generating procedures, high-volume saliva ejectors and 4-handed technique are beneficial for controlling infection.^38,39^

## COVID-19 Transmission Among Dental Personnel

The Centers for Disease Control and Prevention (CDC) update from August 4, 2020, informed that there were no available reports on dental personnel testing positive for COVID-19.^13^ Most dental-related articles released in PubMed provide general information on SARS-CoV-2 outbreak and recommendations for infection control in the dental office. Only 1 article referred to COVID-19 infection among dental personnel.^15^ The information presented comes from the School and Hospital of Stomatology, at Wuhan University, and reports 9 COVID-19 positive persons among 1098 staff. Except for 2 cases, no obvious epidemiological aggregation was identified for the other COVID-19-positive persons and cross-infection was unlikely. No further COVID-19 infection has been reported among staff. Moreover, 169 staff were involved in duty of dental emergency and treated more than 700 patients. No one developed COVID-19, which confirmed the effectiveness of infection control procedures within dental settings.^15^

## The Functioning of Cluj-Napoca, Romania, Dental Emergency Setting During COVID-19 Pandemic

During the national emergency status declared in Romania, a 5-unit dental setting was organized in Cluj-Napoca to treat non-COVID-19 patients. The dental setting functioned for 6 wk (April 3 to May 15) during a period when only 3 other private dental settings were functioning.

Strict circuits for personnel and patients, disinfection, donning and doffing personal protective equipment (PPE) protocols were developed and implemented through effective instruction.

The medical care was provided by 31 dental specialists and more than 200 residents. There were 2 shifts per d, 7 d per week, except Sundays with only 1 shift. The personnel were planned to work in groups of 1 senior dentist and 3 residents per shift, so that a 14-d isolation interval for each medical person could be calculated.

During the above-mentioned period, more than 1000 patients with emergency dental problems were provided effective treatment. All the dental procedures were recorded daily. In addition, 1600 patients received dental phone consultations. Even under the SARS-CoV-2 threat, emergencies continued to be a real problem. In Beijing, China, the overall dental emergency patients count reduced by only 38%, between February 1 and February 10, 2020.^40^

According to the Covid-19 safety planning framework for emergency dentistry interventions, adopted by the Romanian College of Dentists, 17/3/BExN/2020,^41^ only the following pathologies were considered emergencies: tooth pain-related pathologies, dental or periodontal abscesses, pericoronitis, postextractional alveolitis, cellulitis/abscesses, mandibular fractures, temporo-mandibular joint dislocation, dentoalveolar traumas; ulceronecrotic gingivostomatitis.^41^ Traumatic disorders were treated in a surgical establishment.

Protocols were elaborated for each treatment approach and learned by each team member. Aerosol-generating procedures were strictly minimized. Based on our clinical experience during this outbreak, pulp exposure was made under rubber dam isolation and a high-volume saliva ejector after local anesthesia as well as a high-speed handpiece were used most of the time to access cavity preparation. The filling material was zinc oxide eugenol temporary filling due to its higher resistance in comparison with other provisional materials.

## Measures for Preventing SARS-CoV-2 Contamination in Dental Settings

Present recommendations are based on our current experience, on the experience reported by our colleagues,^15^ on systematic reviews^42,43^ and relevant guidelines provided by different authorities such as the US Department of Labor (2020),^44^ the CDC,^13^ and the Association Dentaire Française (2020).^9^ Although these protocols have been designed for an emergency dental practice, they can be extrapolated and adapted by all dental settings, on a long-term basis.

**Figure 1.**
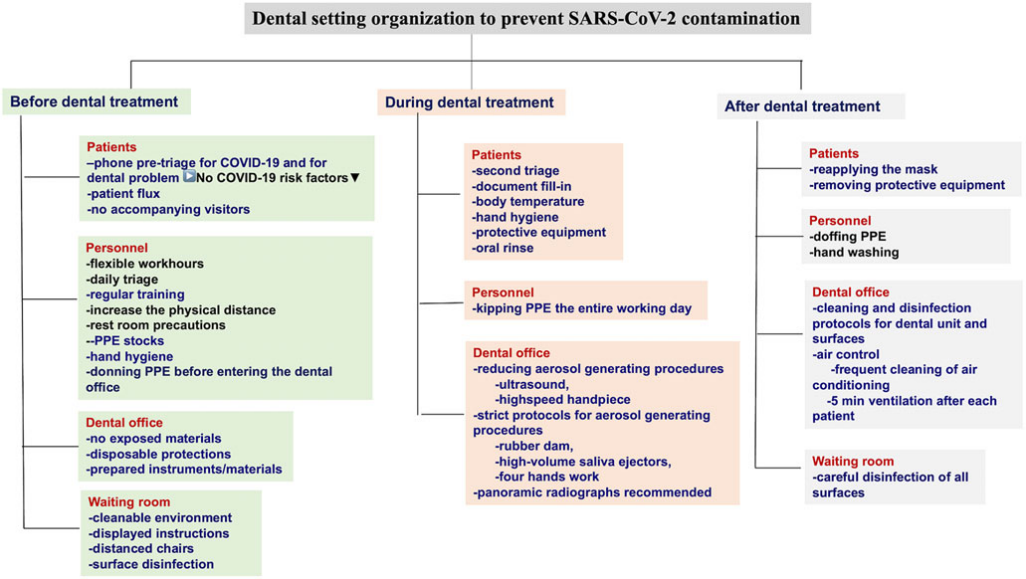
Guideline for dental setting organization to prevent SARS-CoV-2 contamination. Overall view of preventive measures.^13,15,44^

**Figure 2.**
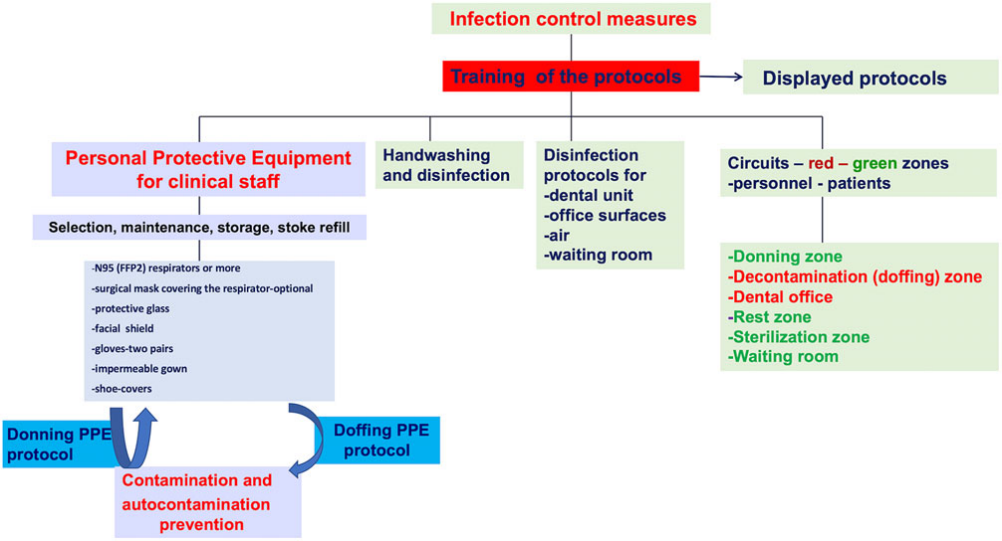
Infection control in the dental setting.^9,15,44^

To prevent SARS-CoV-2 contamination, the function of dental office should rely on administrative and infection control measures (Figures 1 and 2).

### Measures Before Dental Treatment

#### Patients

Precheck phone triage is an important step that decreases the time spent by the patient in the dental office. During the phone triage, potential patients are asked a set of standard clinical questions about the COVID-19 symptoms and contact or travel history to detect any potential risk of SARS-CoV-2 infection. Determining the dental problem is another target of the phone triage.

A dental visit is subsequently scheduled. When unnecessary, no accompanying visitors are advised.^13^ When this is not feasible, the accompanying person is asked to wait outside.^45^

#### Personnel

Organizing flexible work hours, daily personnel triage, wearing a face mask in all dental settings are important aspects of human resource management. The dental team is discouraged from using common electronic office technology or furniture (phones, desks, keyboards).^13^ An essential step is to provide the personnel with adequate information and training, while emphasizing the proper use of PPE (Figure 1). A clear procedure describing the safe sequence of donning and doffing PPE should be provided (Figure 2). Information and training of dental personnel is mandatory to prevent contamination. All protocols should be posted in easily visible places.

Hand-washing should be performed for at least 60 s, using a 60 to 85% hydroalcoholic solution, before dental treatment, before wearing gloves.^45^

Preparation of dental staff for clinic is based on PPE including gloves, goggles, face shields, face masks, and respiratory protection (Figure 2). To be protective, PPE should be properly worn^46^).

As respiratory droplets are the main route of SARS-CoV-2 transmission, particulate respirators such as N-95 masks, authenticated by the US National Institute for Occupational Safety and Health, or FFP2-standard masks set by the European Union are recommended for routine dental practice.^13,15,47^ A N95 respirator filters at least 95% of airborne particles, but it is not resistant to oil.^48^ During disposable N95 filtering facepiece respirators shortages, consider using other respirators that provide greater protection such as R/P95, N/R/P99, or N/R/P100 filtering facepiece respirators or FFP3 masks.^47^

Although disposable respirators, such as N95, are not approved for routine decontamination, due to shortage of stocks, their disinfections and reuse have been discussed.^49^ UV germicidal irradiation and vaporous hydrogen peroxide seem to be extremely promising methods for face filtering respirator decontamination.^46,49,50^ Some shortcomings related to these methods have been reported, especially the paucity of evidence relating to their effectiveness specifically against SARS-CoV-2 and the possible modifications of the filtering capacity.^46,49^

The special respirator shall be worn at all times during a work shift; this is the last PPE to be removed during the doffing procedure. A face full shield may also be worn in front of a respirator to prevent bulk contamination of the respirator and to avoid eye contamination. Protective glasses are also recommended.

A long-sleeve impermeable surgical gown or protective clothing is used to cover personal clothing and skin. A second disposable paper gown is worn over the top. Disposable gowns are discarded after each use. Two pairs of gloves should be used, the exterior pair being removed after each patient.

Clinical staff clothing includes a complete set of PPE: special respirators—at least a N95 or a FFP2, protective glasses, a face shield, an impermeable gown plus a paper gown, gloves, shoes-covers, and a surgical cap (Figure 2). Due to PPE stock shortage, the nonclinical staff members should wear a normal surgical mask, a disposable paper gown over personal clinical clothing, and a face shield.

Personnel doff PPE before entering dental practice (Figure 2).

#### Dental Office

All the necessary materials and instruments should be prepared in advance to make the procedure faster. Disposable protections should be placed on unit surfaces and single-use instruments should be used at all times, whenever possible.

#### Waiting Room

Instructions (in appropriate languages) about hand hygiene, cough etiquette, and facemask coverage during the visit period should be displayed in the waiting room. A minimal number of patients should be allowed in the waiting rooms and distanced chairs should also be considered.^25^

Thorough disinfection of potentially contaminated surfaces such as door handles, chairs, and desks is also strongly reccomended^15,51^ (Figure 1).

### Measures During Dental Treatment

#### Patients

Patients should wear medical masks when entering the waiting room. Body temperature is checked before dental treatment, and the patient performs hand hygiene with a hydroalcoholic solution. A second triage is performed, and patients complete the necessary forms. A single-use gown and shoe-covers are provided for patients to use (Figure 1).

One-minute mouth rinses with 1% hydrogen peroxide are strongly recommended, especially when the rubber dam is not used for the dental procedure.^45^

#### Personnel

PPE is worn at all times during treatment, except for the exterior pair of gloves and the exterior gown which are changed between patients. Face shields are also disinfected. Under no circumstances will the clinical staff remove respirators, or perform the doffing protocol in the dental office (Figure 2).

#### Dental Office

Although highly recommended, the reduction of aerosol generating procedures could be a difficult task. The use of the rubber dam in restorative dentistry and endodontics and the focus on minimally invasive procedures (hand instruments only) in restorative dentistry and periodontology should be prioritized.

High-volume saliva ejectors and 4-hand work are highly recommended^37,38^ to limit atmospheric microbial contamination.^52^

Panoramic radiograph is an appropriate alternative to intraoral radiograph as it minimizes saliva secretion and coughing.^15^

### Measures After Dental Treatment

#### Patients

They reapply the face mask and remove the protective barriers when exiting the dental office (Figure 1).

#### Personnel

At the end of the working day, the clinical staff follow a strict protocol for doffing PPE, that takes place in a special decontamination/doffing room. PPE should be carefully removed, eventually disinfected,^46^ and stored or disposed of, to avoid contamination.^47,53^ Thorough hand washing is performed after the removal of PPE.^45^ There is no evidence to support the effectiveness of sanitizing tunnels in reducing the spread of COVID-19 and they could cause skin, eye, or respiratory irritation or damage.

#### Dental Office

Cleaning and disinfection of the dental office after the exit of a nonconfirmed COVID-19 patient should start 15 min after completion of clinical care. This is recommended to allow for droplets to sufficiently fall from the air after a dental procedure.^54^

The disposable protection of the surfaces is to be removed. Strict disinfection protocols for dental units and for the other office surfaces using the recommended cleaning and disinfection solutions are crucial due to the long persistence of SARS-CoV-2 on surfaces.^22,55^

Shields and glasses are disinfected with 70% isopropyl alcohol.

A 5-min air change is strongly advised after each patient.^45^ Also, air control through frequent disinfection of air conditioning is recommended.^13^ UV decontamination can be used (Figure 1).

#### Waiting Room

Careful disinfection of potentially contaminated surfaces is strongly suggested.^15,51^

During a 45-d period, the supplementary costs of preventive disinfection measures, single-use instruments (high speed handpieces and examination instruments) and PPE upgrades can amount to 16,000 Euros, with 2 shifts per day and 4 medical staff per shift.

## Discussion

The present article provides an overview of the management of SARS-CoV-2 transmission mostly in relation with the dental office, as well as new guidance that will enhance the organization of dental settings to prevent SARS-CoV-2 contamination and brief information on the economic impact of preventive measures in dental settings. The provided protocols are based on former or very recent evidence or draw upon the experience of the team of dental professionals who delivered emergency dental care during COVID-19 lockdown and after the restart of the activity.

According to the US Occupational Safety and Health Act, dentists are considered a very high-risk category for SARS-CoV-2 transmission.^41^ Recent evidence suggests that even nonsymptomatic persons can spread COVID-19 with high efficiency^7,44^ ; thus, every healthy patient has been considered potentially contagious and additional measures beyond standard precautions^15^ have been implemented to limit and combat SARS-CoV-2 contamination in our settings.

Given its high preventive efficiency, our team strongly recommends the implementation of the present detailed guideline. Even after the lockdown period, the same preventive protocol was extended to 3 other settings that were part of the Dental Ambulatory at the Emergency County Clinical Hospital. None of the medical staff developed COVID-19 symptoms, except a nurse who tested positive for SARS-CoV-2 due to family exposure, but remained asymptomatic. Also, 2 residents developed mild symptoms and tested positive during their vacation period. No seropositive staff was reported after random antigen testing of the personnel.

The available literature to date and the actual clinical experience of health-care professionals still fail to establish high-certainty, ideal protective measures that could prevent SARS-CoV-2 contamination in dental settings. In the meantime, the infection control protocols that we, as well as other teams,^15,45,51^ used seemed to be relatively efficient in avoiding SARS-CoV-2 contamination. It is our opinion that the same intensive care equipment and the strict organizational measures used by clinical workers during the emergency care period should become the practice standard for everyday clinical activity. To reduce dental aerosol contamination, dental teams should rely on multiple precautionary strategies such as the use of personal protection barriers, the reduction of aerosol-producing procedures, the use of preprocedural antiseptic rinses and the routine use of a high-volume evacuator. Although inexpensive, the last 2 strategies are rarely used in most dental settings.^19^ The extreme dynamicity of the outbreak and of the related information may lead to a sudden shift in views and recommendations for preventing SARS-CoV-2 contamination.

Beyond the infectious risk, COVID-19 also has important economic consequences due to the extreme protective measures^45^ in place. During the 45-d functioning period of our emergency dental setting, around 10,000 Euro from the total amount was spent on single-use equipment for personal protection, such as special respirators (FFP2 or FFP3), surgical masks, impermeable gowns plus paper gowns, double gloves, shoes-covers, and surgical caps. Thus, in response to additional protective measures, an average of 28 Euro per medical staff per day was spent. It has been reported that 4 sets of PPE (N95 respirators, double gloves, gowns, and goggles) per day was needed for each health-care personnel working in a high-risk respiratory infection group during the H1N1 influenza pandemic.^56^ Over a 2-mo period during the influenza season, the incremental cost to prevent a clinical respiratory illness of health-care personnel with continuous use of N95 respirators when compared with surgical masks ranged from US$490 to US$1230, depending on the type of respirator (fit non-fit) or manufacturer. However, due to limited resources, changing of the respirator during a working day is difficult to conceive. In the case of highly pathogenic pandemic, respirator use for health-care workers would likely be a highly cost-effective intervention.^57^

COVID-19 also has a long-term impact on clinical practice, patients, dental education, and dental research^7^ as well as on dental personnel. It is possible that, from now on, patients could become more careful with their oral health and more likely to adhere to prophylactic measures. An increasing demand for computer aided design and manufacturing (CAD/CAM) and 3D-printing technology is also anticipated.^7^ On the contrary, due to the fear of contamination or to economic difficulties patients might, for instance, prefer extractions over a prolonged root canal treatment. The difficulties associated with dentist visits are a significant factor in the initiation or the recurrence of periodontal disease. Moreover, many team members have reported a higher level of work-related stress and anxiety due to heavy workload, the fear of becoming a SARS-CoV-2 victim, of being an asymptomatic spreader, and the risk of infecting their family members.^10^ With the COVID-19 outbreak, teledentistry became a very effective tool for patient triage, consultations, diagnosis, and patient monitoring. Dentists have also been able to prescribe medication, such as pain killers and antibiotics. Overall, teledentistry proved to be a very efficient way of limiting office visits for patients seeking dental advice, additionally reducing infection-related stress for both patients and doctors.^58^ Teledentistry could also be a very effective way of promoting preventive measures for dental hygiene, reducing the risk of periodontitis and dental disease. Our team initiated a preventive care program directed to periodontitis patients receiving initial therapy or in maintenance phase. Patients received personalized oral hygiene advice from specialists on a virtual platform. However, it is still too early to appreciate the cost-benefit ratio of this approach. Also, the psychological impact of an indirect contact and the consecutive efficiency of teledentistry advice should be further evaluated.

It could eventually be argued that the COVID-19 pandemic marks the beginning of a new era for the organization of dental settings and for the further implementation of infection control measures.

## Conclusions

Dental professionals are highly exposed to the risk of SAR-CoV-2 infection. Due to the unique characteristics of dental procedures, generating a potentially large number of droplets and aerosols, the standard protective measures are insufficient to prevent the spread of COVID-19 in everyday clinical work settings. Considering the high contagiousness of COVID-19, it is important to underline that wearing N95/FFP2 or FFP3 respirators is mandatory to prevent SARS-CoV-2 infection. The underestimation of the persistence of the risk of viral transmission might have disastrous consequences on patients and on the medical personnel.

The implementation of strict preventive measures proved to be efficient in the prevention of SARS-CoV-2 contamination because no infections have been reported among our staff or patients.
